# The magnitude of the pharmacodynamic index for NOSO-502: pathogen clearance, emergence of resistance and human dose predictions

**DOI:** 10.1093/jac/dkag106

**Published:** 2026-03-24

**Authors:** Sanne van den Berg, Marie Attwood, Pippa Griffin, Alan Noel, Shampa Das, Markus Zeitlinger, Emilie Racine, Xavier Boulenc, Anouk E Muller, Sebastiaan D T Sassen, Soma Bahmany, Marian T ten Kate, Alasdair MacGowan, Joseph Meletiadis

**Affiliations:** Department of Medical Microbiology and Infectious Diseases, Erasmus MC University Medical Center, Rotterdam, The Netherlands; CATOR, Center for Antimicrobial Treatment Optimization Rotterdam, Rotterdam, The Netherlands; Bristol Centre for Antimicrobial Research & Evaluation (BCARE), Infection Sciences, Southmead Hospital, Westbury-on-Trym, Bristol, UK; Bristol Centre for Antimicrobial Research & Evaluation (BCARE), Infection Sciences, Southmead Hospital, Westbury-on-Trym, Bristol, UK; Bristol Centre for Antimicrobial Research & Evaluation (BCARE), Infection Sciences, Southmead Hospital, Westbury-on-Trym, Bristol, UK; Antimicrobial Pharmacodynamics and Therapeutics, University of Liverpool, Liverpool, UK; Department of Clinical Pharmacology, Medical University of Vienna, Vienna, Austria; Nosopharm, Nîmes, France; DMPK, Evotec, Lyon, France; Department of Medical Microbiology and Infectious Diseases, Erasmus MC University Medical Center, Rotterdam, The Netherlands; CATOR, Center for Antimicrobial Treatment Optimization Rotterdam, Rotterdam, The Netherlands; Department of Medical Microbiology, Haaglanden MC, The Hague, The Netherlands; CATOR, Center for Antimicrobial Treatment Optimization Rotterdam, Rotterdam, The Netherlands; Department of Hospital Pharmacy, Erasmus MC University Medical Center Rotterdam, Rotterdam, The Netherlands; Department of Hospital Pharmacy, Erasmus MC University Medical Center Rotterdam, Rotterdam, The Netherlands; Department of Medical Microbiology and Infectious Diseases, Erasmus MC University Medical Center, Rotterdam, The Netherlands; Bristol Centre for Antimicrobial Research & Evaluation (BCARE), Infection Sciences, Southmead Hospital, Westbury-on-Trym, Bristol, UK; Department of Medical Microbiology and Infectious Diseases, Erasmus MC University Medical Center, Rotterdam, The Netherlands; Clinical Microbiology Laboratory, Attikon University Hospital, Medical School, National and Kapodistrian University of Athens, Athens, Greece

## Abstract

**Background:**

NOSO-5O2 is the first clinical candidate of a new antimicrobial class, the odilorhabdins. The pharmacodynamics of NOSO-502 was studied to establish the magnitude of the pharmacodynamic index (PDI) and make human dose predictions.

**Methods:**

*In vitro* experiments using different types of media were performed in time–kill curves and a pharmacokinetic model. *In vivo* experiments were conducted in the neutropenic murine thigh infection model. Six *E. coli* (MIC 1–8 mg/L) and two *K. pneumoniae* (MIC 1–2 mg/L) strains were used. 24 h bacteriostatic and 1- and 2-log_10_ kill effects were related to *f*AUC_0–24_/MIC and *f*AUC_0–24_/MIC per length of dosing interval (*f*AUC_0–24_/MIC·1/tau). Human pharmacokinetic parameters were predicted using interspecies allometric scaling and used to simulate the dose needed to reach the bacteriostatic PDI target for *E. coli*.

**Results:**

The *in vitro* activity of NOSO-502 was dependent on the media and the strength of Mueller–Hinton Broth II (MHBII) used such that *f*AUC_0–24_/MIC ratios were higher when measured in 100% MHBII than 50% MHBII. *In vivo* for *E. coli*, the *f*AUC_0–24_/MIC for bacteriostatic effect and 1-log_10_ reduction in bacterial count were 10.7 ± 10.9 and 18.2 ± 16.5, respectively. The final human predicted parameters of the model had CV values of <20%. The human dose required to achieve the bacteriostatic *f*AUC_0–24_/MIC for each *E. coli* strain varied from 149 to 1717 mg/day.

**Conclusions:**

A combination of the use of PDI targets and prediction of human pharmacokinetics allowed effective doses of NOSO-502 in man to be estimated.

## Introduction

Enterobacterales, such as *Escherichia coli* and *Klebsiella pneumoniae*, have the dubious honour of leading the list of antibiotic-resistant bacterial priority pathogens published by the WHO.^1^ This most critical group of pathogens includes multidrug-resistant bacteria that pose a threat in hospitals, nursing homes and among patients who are dependent on devices such as intravenous catheters, prosthetic implants and ventilators. These pathogens can cause severe and often deadly infections and have become resistant to many antibiotics. The development of new antimicrobials is key to fight the increasing antibiotic resistance in such high-grade pathogens,^2^ which was estimated to have resulted in 1.27 million deaths in 2019,^3^ with numbers rising.

Odilorhabdins are a novel class of peptide antibiotics that bind to the decoding centre of the 16S subunit of the bacterial ribosome at a site not exploited by any known ribosome-targeting antibiotic.^4^ A lead optimization programme using medicinal chemistry has led to the development of promising lead odilorhabdin candidate, NOSO-502. Its antimicrobial activity against *E. coli* and *K. pneumoniae* has been shown both in *in vitro* time–kill experiments and in murine infection models.^5^ Its pharmacodynamics (PD) have been studied in the neutropenic murine thigh infection model with *E. coli* and *K. pneumoniae* strains, which suggested *f*AUC/MIC as the dominant PD index (PDI).^6^ In our recent study,^7^ we have shown in the same murine model as well as in the *in vitro* pharmacokinetic model that NOSO-502 efficacy was best described by the *f*AUC/MIC but also time of drug exposure.^8^ In the present study, we determined the magnitude of both *f*AUC_0–24_/MIC and a modified pharmacodynamic index which incorporates both AUC and time of exposure (*f*AUC_0–24_/MIC·1/tau) which were associated with stasis and bactericidal outcomes in an *in vitro* pharmacokinetic and neutropenic murine thigh infection model. These values were subsequently used to make predictions of the effective human dose.

## Materials and methods

### Bacteria, media and antibiotics

Six *E. coli* and two *K. pneumoniae* isolates were used for these studies (Table S1, available as Supplementary data at *JAC* Online). The MICs of NOSO-502 and eight commonly used antibiotics against the bacterial isolates used in this study were determined before by broth microdilution according to the ISO guidelines.^9,10^ Strains were representatives of the NOSO-502 MIC range 1–8 mg/L, MIC_90_ 4 mg/L against *E. coli*, and varying resistance patterns including ATCC wild-type isolates.^10^ Isolates grew well in *in vitro* and *in vivo* infection models. Cation adjusted Mueller–Hinton broth II (MHBII, BBL Le Pont de Claix, France) and agar (MHA, ThermoFisher, UK) was used for subculturing and quantification. Nosopharm SAS (Nîmes, France) supplied NOSO-502. The compound was reconstituted in sterile phosphate buffer (pH 7.4). All dose levels are presented as net peptide.

### *In vitro* time–kill curves in different media

Overnight cultures of *E. coli* ATCC 25922 were diluted into fresh 100% MHBII, 50% MHBII, RPMI and DMEM to yield an inoculum of 1 × 10^6^ cfu/mL in a total volume of 10 mL. Drug was added at 0, 5, 10, 15 and 30 mg/L and cultures were incubated at 37°C in air for 24 h. One millilitre samples were taken at *t*= 0, 2, 4, 6, 8, 12, 24 h; aliquots (100 μL of neat and 10^−3^ dilution) were plated onto nutrient agar plates using a spiral plater (Don Whitley Scientific, Yorkshire, UK) and incubated for 24 h before counting.

### *In vitro* pharmacokinetic model

A Fermac 301 Fermentation System (Electrolab, Tewkesbury, UK) *in vitro* PK model was used to simulate concentrations of NOSO-502 administered every 8 h as described previously.^7^ The model has been validated in comparison to other *in vitro* pharmacokinetic models^11^ and a fuller description is given in MacGowan *et al*.^12^ NOSO-502 exposures were simulated using a *t*_1/2_ of 4 h and *C*_max_ up to 24.1 mg/L. Two isolates (*E. coli* ATCC 25922 and *K. pneumoniae* 700603) were initially tested and PK/PD targets were subsequently determined for another two *E. coli* strains. Samples were taken throughout the sampling period up to 24 h for quantification of viable counts. Bacteria were counted by a spiral plater (Don Whitley Systems, Shipley, West Yorkshire, UK). Aliquots were also taken at 0, 24 and 48 h and plated on to nutrient agar plates containing 4× and 8× NOSO-502 MIC to study emergence of resistance. Plates were incubated for a minimum of 18 h in air at 36°C and the viable counts determined. The minimum level of detection was 10^2^ cfu/mL. Aliquots were stored at −70°C for NOSO-502 assay.

### Ethical approval

All pre-clinical studies described in this study were conducted in accordance with the recommendations of the European Community (EU Animal Directive 2010/63/EU 2010 directive) and approved by the Institutional Animal Care and Use Committees of Erasmus MC (IRN 2019–0018) and Aptuit Committee on Animal Research and Ethics (Experimental Project internal code: 36101).

### *In vivo* thigh infection model

Female outbred CD-1 mice (Charles River Germany), 7–8 weeks old, weight (mean ± SD) 25.0 ± 1.5 g, specified pathogen-free, were used. These were housed under standard conditions with food and water supplied *ad libitum* and allowed to acclimatize at least 1 week in the facility.

NOSO-502 efficacy was determined in the neutropenic thigh infection model as described before.^7^ Briefly, neutropenic mice were infected with 6–60 × 10^5^ cfu/50 µL in each lateral thigh muscle using *E. coli* strain ATCC 25922, C1.4, C1.7, C1.94 or C1.40, or *K. pneumoniae* strain ATCC 43816 (one strain in both thighs). NOSO-502 was dosed subcutaneously every 6 h, starting 2 h after infection, 11–352 mg/kg (*E. coli* ATCC 25922, C1.4, C1.7), 22–705 mg/kg (*E. coli* C1.94, C1.40) or 5.5–705 mg/kg (*K. pneumoniae* ATCC 43816) net peptide total daily dose, three mice per dosing group. Phosphate buffer was used as placebo treatment. Mice were randomly allocated to experimental groups. At *t* = 24 h, mice were humanely killed, unless the welfare of the animals indicated earlier termination was necessary, following animal welfare regulations. Bacterial load in thigh muscles at *t* = 0 and *t* = 24 h was determined, and expressed as the difference between the log_10_[cfu/thigh] values ‘Δlog_10_ cfu’.

Murine NOSO-502 plasma levels were determined using LC–MS/MS analysis as previously described.^7^ Free plasma levels were calculated based on 83% plasma protein binding, and PK was described in a population PK model.

### PK/PD analyses

The *in vitro* and *in vivo* exposure–response relationships Δlog_10_ cfu versus *f*AUC_0–24_/MIC and *f*AUC_0–24_/MIC·1/tau were quantified and the relationships were analysed with nonlinear regression analysis using the sigmoid *E*_max_ (hill) model (GraphPad Prism v.8.0, San Diego, CA, USA). The PK/PD indices required for a static, 1- and 2-log_10_ kill effect for *E. coli* and *K. pneumoniae* isolates were determined. The coefficient of determination (*R*^2^) from this model was used to numerically quantify the strength of this relationship.

### Human dose prediction

Human pharmacokinetic parameters of NOSO-502 (Cl, Vd_ss_, *t*_1/2_) were assessed using interspecies allometric scaling of the observed pharmacokinetic parameters from animal studies.

Mouse, rat, dog and non-human primate PK parameters were factorized to predict human PK parameters, as described hereafter for each PK parameter.

In addition, the human PK profile was predicted using the Wajima method, on the basis of the normalized PK profile of each species.^13^

The prediction of clearance (Cl) and volume of distribution at steady state (Vd_ss_) can be achieved by either scaling from animal PK data or *in vitro* microsomal/hepatocyte Cl (that is, *in vitro–in vivo* extrapolation, IVIVE).^14,15^ NOSO-502 was found to be metabolized in blood and hepatocytes *in vitro* in human and pre-clinical species.^5^ However, because hydrolases are ubiquitous, it is likely that multiple organs are responsible of the clearance of the compound. Thereby, no IVIVE was investigated to predict human clearance. Three methods were selected for clearance prediction: simple allometry, simple allometry with Fup correction and fraction unbound corrected intercept (i.e. FCIM) correction.

Three methods were selected for Vd_ss_ prediction: Oie–Tozer, allometry with plasma fraction unbound (i.e. Fup) correction and individual species extrapolation with Fup correction. For both, Vd_ss_ and Clearance, the methods selected are fully described elsewhere.^14–17^

Sophisticated human dose projection requires the prediction of time–concentration profile targeting *C*_max_ or *C*_trough_ to achieve the desired efficacy, in addition to the human PK parameters (see previous paragraph). A widely adopted approach was proposed by Wajima *et al.*^13^ using intravenous (i.v.) predicted human PK parameters (Cl and Vd_ss_) and the time–concentration profiles generated from pre-clinical animal species. This approach yields a normalized time–concentration plot with new combined (dimensionless) data points by dividing the concentration by steady-state concentration (*C*_ss_; dose/Vd_ss_) and the time by mean residence time (Vd_ss_/Cl), respectively, for each species. By so doing, one predicted human PK profile is generated for each pre-clinical species, as described in Lombardo *et al.*^14^ and Vuppugalla *et al*.^17^ However, if the profiles are not fully superimposed, they reflect uncertainty in the method. All normalized humanized PK profile were fitted in one shot in Phoenix software with naïve pooled data option to estimate the average human PK parameters, and their associated uncertainty (reflected by the CV) from all the PK profile (Phoenix Certara, NLME version 8.3.5.340). The naïve pooled data function, when applied to population data, treats all observations as if they came from a single individual (average human in this case) in that it ignores inter-individual variations.

PK human parameters were then used to simulate the appropriate doses (for a human body weight of 70 kg) to assess the dose needed to reach *f*AUC_0–24_/MIC PDI target for each strain, previously assessed in the neutropenic murine thigh infection model.

## Results

### *In vitro* dose responses

**Figure 1.**
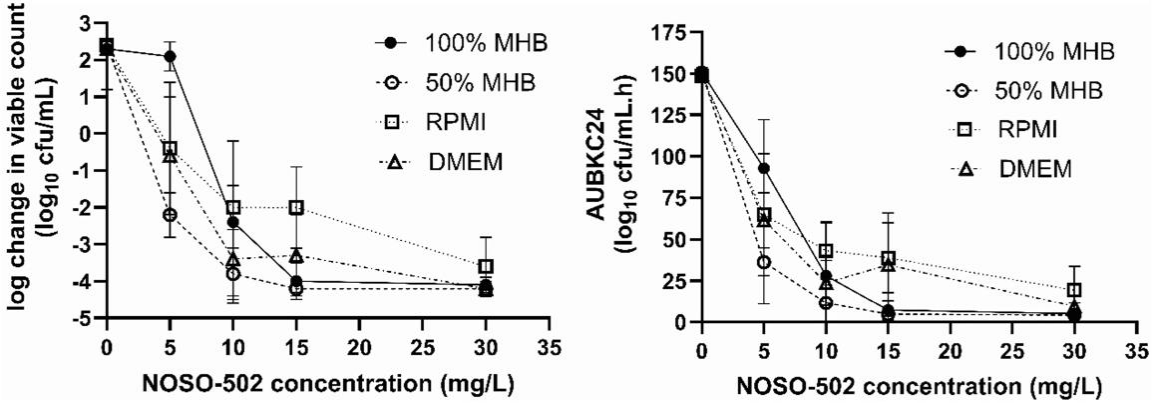
Effect of 100% MHB, 50% MHB, RPMI and DMEM on the activity of NOSO-502 against *E. coli* ATCC 25922 (means and standard deviations are shown).

Time–kill curves with *E. coli* ATCC 25922 across a range of concentrations of NOSO-502 in different media are shown on Figure 1. The strength of MHB used impacted on bacterial kill with greater kill found in 50% MHB compared with 100% MHB particularly at low NOSO-502 concentrations e.g. 5 mg/L resulted in −2, −0.5, −0.6 and +2 log­_10_ cfu/mL change from initial inoculum in 50% MHB, RPMI, DMEM and 100% MHB, respectively.

In the *in vitro* pharmacokinetic model, exposure–response experiments in 50% and 100% MHB with *E. coli* ATCC 25922 and *K. pneumoniae* 700603 showed that PK/PD targets for stasis, 1-log_10_ and 2-log_10_ kill were lower in 50% MHB compared with 100% MHB particularly for *E. coli* (11.7, 17.7 and 25.2 versus 90.4, 109.6 and 130.3, respectively) and less for *K. pneumoniae* (16.2, 21.7 and 27.8 and 19.3, 34.0 and 56.3, respectively) (Figure S1). Subsequently three strains of *E. coli* and *K. pneumoniae* 700603 strains were tested in the *in vitro* infection model with 50% MHB and the *f*AUC_0–24_/MIC ratios for stasis, 1-log_10_ kill and 2-log _10_ kill together with bacterial load in controls and maximum kill are shown in Table 1. For all the strains a static, 1-log_10_ kill and 2-log _10_ kill effect was achieved at mean *f*AUC_0–24_/MIC 26.7, 47.4 and 85 for *E. coli* and 16.2, 21.7 and 27.8 for the *K. pneumoniae*. The *f*AUC_0–24_/MIC·1/tau were not calculated as in all the exposure ranging experiments NOSO-502 was given 8 hourly.

**Table 1.** NOSO-502 PK/PD fAUC_0–24_/MIC targets associated with net stasis, 1-log_10_ kill and 2-log_10_ kill in the *in vitro* model for *E. coli* and *K. pneumoniae* using 50% MHBII

Species	Isolate or data measure	MIC (mg/L)	Mean ± SD bacterial burden at start of exposure (log_10_ cfu/mL)	Mean ± SD increase of bacterial burden (log_10_ cfu/mL) with 24 h growth in growth controls	Mean ± SD maximum kill at the end of exposure (log_10_ cfu/mL)	*f*AUC/MIC target for stasis	*f*AUC/MIC target for 1-log_10_ kill	*f*AUC/MIC target for 2-log_10_ kill
*E. coli*	ATCC 25922	1	6.0 ± 0.1	2.6 ± 0.1	>−4.2	11.7	17.7	25.2
	C1.94	4	6.5 ± 0.1	2.1 ± 0.1	−3.8 ± 0.1	31.0	51.0	83.0
	1135	1	6.1 ± 0.1	2.5 ± 0.1	>−4.2	37.5	73.4	146.8
	Mean		6.2	2.4	NC	26.7	47.4	85.0
	Median		6.2	2.5	>−4.2	31.0	51.0	83.0
	SD		0.2	0.2	NC	13.4	28.0	60.8
*K. pneumoniae*	ATCC 700603	2	6.3 ± 0.1	2.0 ± 0.1	−4.2 ± 0.1	16.2	21.7	27.8

NC, Not calculated.

Population profile changes at *f*AUC_0–24_/MIC ratios from 0 to >35 for *E. coli* and 0 to >45 for *K. pneumoniae* are shown in Table S2. Suppression of population changes occurred at *f*AUC_0–24_/MIC of ≥25 for *E. coli* and ≥45 for *K. pneumoniae*.

### *In vivo* dose responses

In thigh infected mice, at start of treatment, the average bacterial thigh load was 7.05 (range 6.35–7.70) and 7.93 log_10_ cfu for *E. coli* and *K. pneumoniae*, respectively. In placebo treated mice, bacteria grew on average 2.51 (range 1.84–3.08) and 1.55 log_10_ cfu/thigh in *E. coli* and *K. pneumoniae* infected mice, respectively (Table 2).

**Table 2.** NOSO-502 PK/PD fAUC_0–24_/MIC and fAUC_0–24_/MIC·1/tau targets associated with net stasis and 1-log_10_ kill in the neutropenic murine thigh infection model for *E. coli* and *K. pneumoniae*

Species	Isolate or data measure	MIC (mg/L)	Mean ± SD bacterial burden at start of treatment (log_10_ cfu/thigh)	Mean ± SD increase of bacterial burden (log_10_ cfu/thigh) with 24 h growth in placebo treated controls	Mean ± SD maximum kill at the end of therapy (log_10_ cfu/thigh)	Targets for stasis		Targets for 1-log_10_ kill	
						*f*AUC/MIC	*f*AUC/MIC·1/tau	*f*AUC/MIC	*f*AUC/MIC·1/tau
*E. coli*	ATCC 25922	1	7.70 ± 0.16	1.84 ± 0.19	−2.85 ± 0.59	17.99	3.00	28.48	4.75
	C1.4	1	6.85 ± 0.12	3.08 ± 0.30	−1.74 ± 0.13	4.37	0.73	11.67	1.94
	C1.7	2	7.34 ± 0.04	2.29 ± 0.21	−0.96 ± 0.20	25.11	4.19	41.82	6.97
	C1.94	4	6.35 ± 0.11	3.06 ± 0.28	−1.38 ± 0.96	1.50	0.25	5.81	0.97
	C1.40	8	7.02 ± 0.04	2.25 ± 0.10	−1.34 ± 0.14	1.36	0.23	2.99	0.50
	Mean		7.05	2.51	−1.60	10.07	1.68	18.16	3.03
	Median		7.04	2.39	−1.30	4.37	0.73	11.67	1.94
	SD		0.46	0.52	0.73	10.85	1.81	16.52	2.75
*K. pneumoniae*	ATCC 43816	1	7.93 ± 0.12	1.55 ± 0.57	−1.80 ± 0.15	2.95	4.00	0.49	0.67

**Figure 2.**
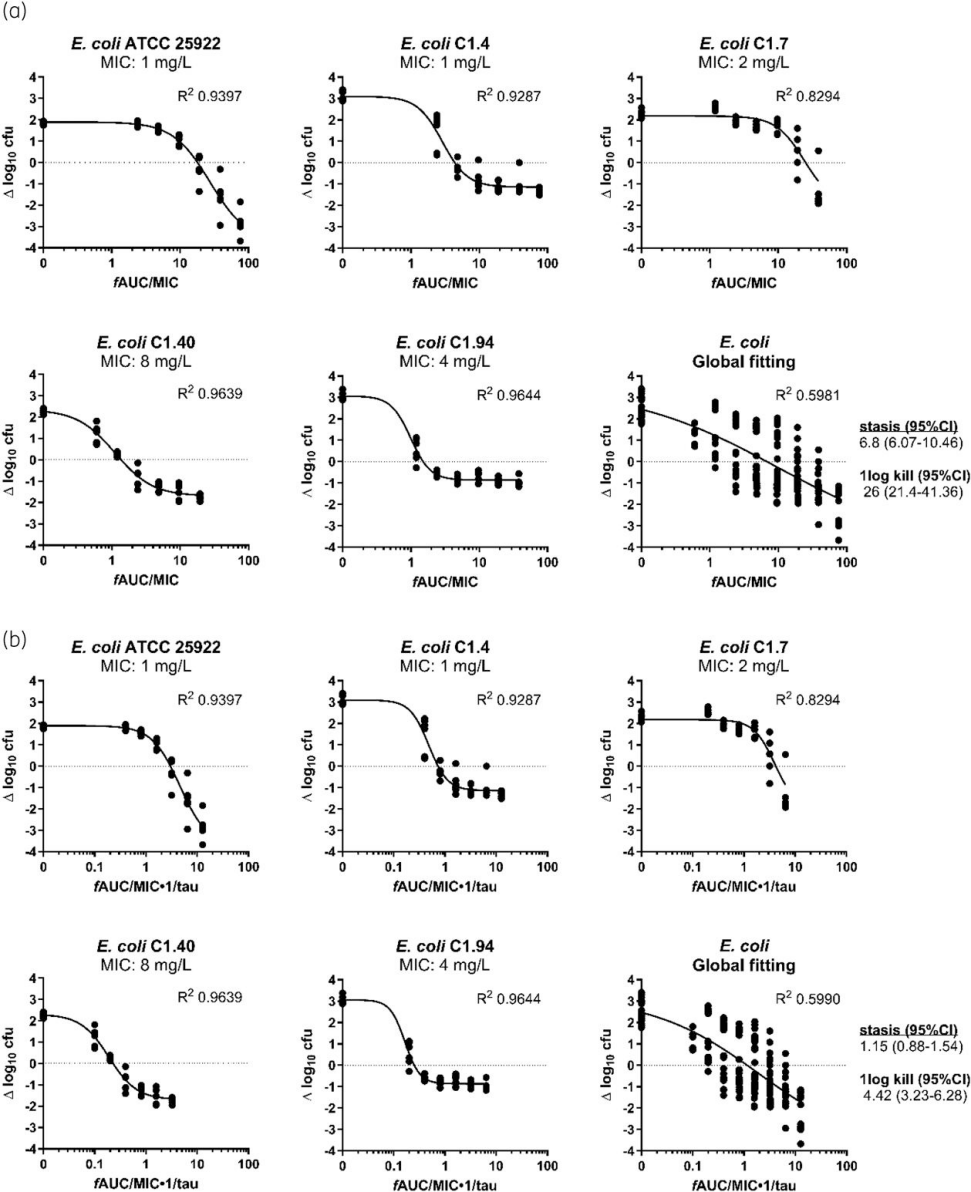
Exposure–response relationships of NOSO-502 *f*AUC_0–24_/MIC (a) and *f*AUC_0–24_/MIC·1/tau (b) for each strain individually and for all strains together. Each dot represents a therapy response in one mouse thigh. The line is the best-fit line based on the sigmoidal *E*_max_ model.

For all isolates, a static and 1-log_10_ kill effect was achieved; for two of six strains (*E. coli* ATCC 25922 and *K. pneumoniae* ATCC 43816), a 2-log_10_ kill effect was observed at the doses tested. The *E*_max_ model well described the exposure–response relationship of all isolates, and the static and 1-log_10_ kill *f*AUC_0–24_/MIC and *f*AUC_0–24_/MIC·1/tau targets were calculated (Table 2, Figure 2). For *E. coli* (five strains), the mean *f*AUC_0–24_/MIC targets associated with stasis and 1-log_10_ kill were 10.07 and 18.16, and *f*AUC_0–24_/MIC·1/tau targets associated with stasis and 1-log_10_ kill were 1.68 and 3.03, respectively. For *K. pneumoniae* (1 strain), the *f*AUC_0–24_/MIC target associated with stasis and 1-log_10_ kill were 2.95 and 4.00, and *f*AUC_0–24_/MIC·1/tau targets associated with stasis and 1-log_10_ kill were 0.49 and 0.67, respectively.

### Human dose prediction

Pre-clinical PK parameters in mice, rats, dogs and non-human primates (cynomolgus monkeys) are shown in the Table S3. NOSO-502 is moderately bound to plasma proteins, with no major species differences. The maximum difference is observed in rat 0.13 versus human 0.25 (Table S4). Blood plasma partitioning ratios (B/P ratios) are consistent between the species. B/P ratios being low, reflecting a low distribution in blood cells (Table S5).

Human PK predicted Vd_ss_ and Cl parameters are shown in Tables S6 and S7, and Figure S2. The methods used exhibit a good consistency to each other for both Vd_ss_ (0.5–0.9 L/kg, overall predicted values 0.7 L/kg) and Cl (14–19.2 L/h, overall predicted value 16.5 L/h). The average values were used as input parameters to generate the human PK parameter prediction (Wajima method). Combining predicted human clearance and volume of distribution, a humanized PK profile was generated for each pre-clinical species. A two-compartment model was used to fit the four PK profiles, through the naïve pooled data option in Phoenix software. Observed versus predicted NOSO-502 individual concentrations are shown in Figure S3, indicating an appropriate fitting of the individual PK profiles.

**Figure 3.**
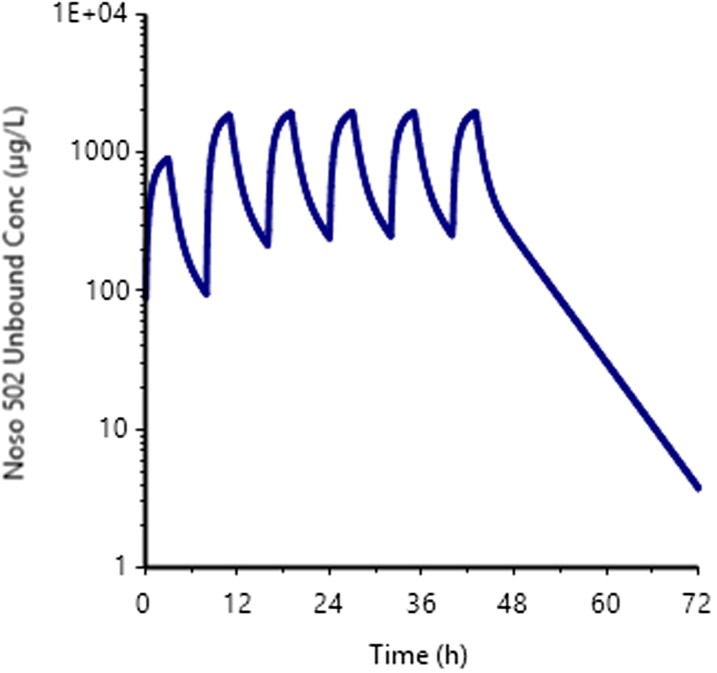
Simulated human PK plasma profile (unbound concentrations) after six repeated administrations of a 372 mg daily dose. This dose is the median predicted human dose required to attain the *in vivo* static PK/PD target, as presented in Table 4.

Predicted human PK parameters, with their associated CV are reported in Table 3, showing a reasonable level of uncertainty reflected by a narrow CV (<20%). These human PK parameters were used to simulate PK profiles at different doses after 3 h infusion in human for a body weight of 70 kg, as exemplified in Figure 3 after 372 daily mg (median value from Table 4), every 8 h for six administrations. A non-compartmental analysis was conducted to assess the size of the exposure parameter (that is, *f*AUC at steady state), for a given dose, to reach the 24 h bacteriostatic *f*AUC/MIC target (Table 2), for each *E. coli* strain (Table 4). Table 2 presents the target *f*AUC to be reached and the corresponding daily dose.

**Table 3.** Final human predicted PK parameters of the model and their associated CV

Parameter	Estimate	CV%
V (L)	21.8	19
V2 (L)	28.8	8.9
Cl (L/h)	17	7.8
Cl2 (L/h)	8.1	12

**Table 4.** Predicted human doses and *f*AUC_0–24_s required to attain the *in vivo* static PK/PD target for the *E. coli* strains tested in animals

*E. coli* strain	MIC (mg/L)	*f*AUC/MIC (mg.h/L) for stasis	Daily dose (mg)	*f*AUC (mg.h/L)
ATCC 25922	1	17.99	615	17.99
C1.4	1	4.37	149	4.37
C1.7	2	25.11	1717	50.20
C1.40	8	1.36	372	10.88
C1.94	4	1.50	205	6.00
Mean			612	17.89
Median			372	10.88

## Discussion

Pre-clinical studies to establish the pharmacokinetic/pharmacodynamic (PK/PD) characteristics of developmental antibacterials are an essential step in establishing effective dosing regimens for clinical trials and ultimately use in infected patients. This is especially the case with classes of drug that have not been previously studied in man.^18^ In this regard, use of several PK/PD modelling approaches is important in understanding how established models can be used to assess drugs with previously unexploited modes of action. NOSO-502, a novel peptide antimicrobial, as the lead compound of the odilorhabdin class, is such an example.

The pharmacodynamics of NOSO-502 against Enterobacterales have been previously studied *in vivo* by Zhao *et al*.^6^ and *in vitro* and *in vivo* by Van den Berg *et al*.^7^ In both these studies *f*AUC/MIC was the dominant pharmacodynamic index although Van den Berg *et al*. also described a time dependent element to NOSO-502s antibacterial effect, which would imply multiple dosing in a 24 h period would be preferred in human therapy.

*In vitro* evaluation of NOSO-502 killing effect and the magnitude of the *f*AUC_0–24_/MIC to produce bacteriostatic and bactericidal effects against *E. coli* and *K. pneumoniae* using time–kill curves and a pharmacokinetic model indicated that the antibacterial effect of NOSO-502 was dependent on the media used being greater in RPMI and DMEM than MHBII and greater in 50% MHBII than full strength. This is probably related to inhibitory peptones in MHBII that antagonize the activity of peptide antimicrobials such as NOSO-502.^19,20^ However, the use of alternative media must be balanced by their ability to sustain bacterial growth. Here we selected 50% MHBII as the media, which allowed the growth of *E. coli* and *K. pneumoniae* strains while partly negating the inhibitory effect of 100% MHBII. This was partly successful in that *f*AUC_0–24_/MIC targets for *E. coli* ATCC 25922 were similar *in vitro* and *in vivo* but not for *E. coli* strain C1.94.

Using six strains of *E. coli*, Zhao *et al*.^6^ reported *f*AUC_0–24_/MIC values of 10.4 ± 6.32 for static effect but were not able to quantify the exposures for a 1-log_10_ kill. Here we found that for 4 *E. coli* strains the *f*AUC_0–24_/MIC for stasis was 10.7 ± 10.9 and for 1-log_10_ kill 18.2 ± 16.5, therefore there was good agreement in terms of *f*AUC_0–24_/MIC static effect target and also in the variability between *E. coli* strains tested. Zhao *et al*.^6^ also reported *f*AUC_0–24_/MIC static effect targets for *K. pneumoniae* to be 4.22 ± 4.73 (range 0.75–12.69), which was comparable to the single strain we tested *in vitro* and *in vivo* (*f*AUC_0–24_/MIC static effect target of 16.2 *in vitro* and 2.95 *in vivo*). The *f*AUC_0–24_/MIC values for each strain were integrated with predicted human pharmacokinetic (PK) parameters—estimated using allometric scaling and Wajima-derived PK profiles—to determine an effective human dose for each strain. The predicted human doses, administered three times daily with a 3-hour infusion, range from 149 mg (*E. coli* C1.4) to 1717 mg (*E. coli* C1.7) per day.

We elected to translate the *E. coli in vivo f*AUC_0–24_/MIC static effect targets into the simulations using the predicted human pharmacokinetics as there was good agreement on the mean target values from the two murine studies.^6,7^ It could be argued that a 1-log_10_ kill was more appropriate, but the potential target was much less clear. The results indicated that the worst-case human dose of NOSO-502 would be 1717 mg as daily dose, for *E. coli* strain C1.7 (*f*AUC_0–24_/MIC for static effect: 25.7) which has a higher *f*AUC_0–24_/MIC value than any of the other strains here or by Zhao *et al*.^6^

This study has some clear limitations, perhaps most notable is the lack of data on *Klebsiella* species where only one strain was tested *in vitro* and *in vivo* so the size of the *f*AUC_0–24_/MIC could not be safely established. As *Klebsiella* species are often multidrug-resistant this data gap will need to be addressed. In addition, though the risk of emergence of resistance was partly studied in some *in vitro* simulations, further work is required. Moreover, we did not use positive controls (active antibiotics) in our studies, which could have provided an additional layer of validation. Nevertheless, both models have been repeatedly used in previous research,^7^ consistently demonstrating their validity. Finally, in the human dose simulations inter-individual human pharmacokinetic differences were not included. Such data will become available with the conduct of Phase 1 studies and strengthened in Phase 2 trials when infection in man is treated.

In conclusion, the data presented here add to that already described for NOSO-502 in terms of its PK/PD characteristics.^6,7^ By establishing the target size of the *f*AUC_0–24_/MIC for static effect and translating this into predicted human pharmacokinetics, based on animal data, we were able to estimate likely effective doses in human. Such information is essential in the evaluation of drug safety in first-in-human Phase 1 studies.

## Supplementary Material

dkag106_Supplementary_Data

## Supplementary data

Figures S1 to S3 and Tables S1 to S7 are available as Supplementary data at *JAC* Online.
