# Supplementary material for: The magnitude of the pharmacodynamic index for NOSO-502: pathogen clearance, emergence of resistance and human dose predictions
Source: J Antimicrob Chemother. 2026 Mar 24;81(4):dkag106. doi: 10.1093/jac/dkag106 (PMC13008829; doi:10.1093/jac/dkag106)

**Supplementary Material**

**Table S1.** Bacterial isolates used in this study.

| **Antimicrobial** | **MIC (mg/L)** | | | | | | | |
| --- | --- | --- | --- | --- | --- | --- | --- | --- |
|  | ***E. coli*** | | | | | | ***K. pneumoniae*** | |
|  | **ATCC 25922** | **C1.4** | **C1.7** | **C1.94** | **C1.40** | **1135** | **ATCC 43816** | **ATCC 700603** |
| **NOSO-502** | 1 | 1 | 2 | 4 | 8 | 1 | 1 | 2 |
| **ampicillin** | 8 | >64 | 4 | >32 | 4 | >32 | 16 | >64 |
| **co-amoxiclav** | 4 | >128/4 | 8/4 | >32 | 8/4 | >32 | ≤2 | 8 |
| **piperacillin/ tazobactam** | ≤4 | 8/4 | 2/4 | 4 | 2/4 | ≤4 | 4 | 16 |
| **ceftazidime** | ≤0.12 | 0.12 | 0.12 | >32 | 0.12 | 0.5 | 0.25 | 32 |
| **meropenem** | ≤0.25 | 0.015 | 0.03 | ≤0.25 | 0.03 | ≤0.25 | ≤0.25 | ≤0.25 |
| **gentamicin** | ≤1 | 0.5 | 0.5 | >16 | 1 | >16 | ≤1 | 8 |
| **amikacin** | ≤2 | 0.5 | 2 | ≤2 | 4 | ≤2 | ≤2 | ≤2 |
| **ciprofloxacin** | ≤0.25 | 0.008 | 0.015 | >4 | 0.5 | >4 | ≤0.25 | 0.5 |

**Table S2.** Population changes for *E. coli* ATCC 25922 and *K. pneumoniae* 700603 after 24 h exposure to NOSO-502.

| **AUC/MIC** | **MICx4 recovery plates** | |  | **MICx8 recovery plates** | |
| --- | --- | --- | --- | --- | --- |
|  | **Simulations with growth / simulations performed** | **Bacterial count (log_10_ cfu/mL)** |  | **Simulations with growth / simulations performed** | **Bacterial count (log_10_ cfu/mL)** |
| ***E. coli*** |  |  |  |  |  |
| **0** | 1 / 2 | 3.2 |  | 0 / 2 | <2 |
| **5** | 1 / 1 | 8.2 |  | 1 / 1 | 2.6 |
| **15** | 1 / 1 | 2.3 |  | 0 / 1 | <2 |
| **25-35** | 0 / 12 | <2 |  | 0 / 12 | <2 |
| **>35** | 0 / 11 | <2 |  | 0 / 11 | <2 |
| ***K. pneumoniae*** |  |  |  |  |  |
| **0** | 0 / 2 | <2 |  | 0 / 2 | <2 |
| **5-15** | 4 / 4 | 6.6±2.2 |  | 1 / 4 | 3.6 |
| **25-35** | 1 / 4 | 2.1 |  | 0 / 4 | <2 |
| **>45** | 0 / 8 | <2 |  | 0 / 8 | <2 |

Table S3. *In vivo* PK parameters in preclinical species after i.v. bolus administration.

| **Species** | **Dose (mg/kg)** | **Vd_ss_ (L/kg)** | **Cl (mL/min/kg)** | **t_1/2_ (h)** |
| --- | --- | --- | --- | --- |
| **Mouse** | 3 | 1.2 | 21.0 | 1.02 |
| **Rat** | 10 | 0.5 | 14.7 | 0.45 |
| **Dog** | 4.5 | 0.3 | 4.1 | 1.3 |
| **NHP** | 10 | 1.1 | 10 | 1.3 |

Table S4. Unbound fraction in plasma (Fup) values in preclinical species and human study.

| **Concentration (ng/mL)** | **Mean unbound fraction ± SD** | | | |
| --- | --- | --- | --- | --- |
|  | **Mouse** | **Rat** | **Dog** | **Human** |
| **1,000** | 0.14 ± 0.08 | 0.13 ± 0.02 | 0.18 ± 0.01 | 0.17 ± 0.07 |
| **10,000** | 0.19 ± 0.01 | 0.14 ± 0.03 | 0.16 ± 0.02 | 0.21 ± 0.04 |
| **100,000** | 0.18 ± 0.04 | 0.13 ± 0.03 | 0.15 ± 0.02 | 0.25 ± 0.02 |

Table S5. *In vitro* blood plasma partitioning (B/P ratios) in preclinical species and human.

| **Concentration (ng/mL)** | **B/P ratio** | | | |
| --- | --- | --- | --- | --- |
|  | **Mouse** | **Rat** | **Dog** | **Human** |
| **1,000** | 0.48 | 0.37 | 0.40 | 0.86; 0.80; 0.74 |
| **10,000** | 0.51 | 0.41 | 0.47 | 0.42; 0.38; 0.45 |
| **100,000** | 0.37 | 0.36 | 0.43 | 0.42; 0.45; 0.47 |

Table S6. NOSO-502 estimated human volume of distribution at steady state (Vd_ss_).

| **Method** | **Estimated volume of distribution (L/kg)** |
| --- | --- |
| **Oie & Tozer** | 0.7 |
| **Allometry with Fup correction** | 0.5 |
| **Individual Species Scaling*** | 0.9 |
| **Overall Predicted Value** | **0.7** |

*: mean of mouse, rat, dog and monkey

**Table S7.** Summary of the predicted human clearance values with different methods (Cl).

| **Method** | **Estimated systemic clearance (L/h)** |
| --- | --- |
| **Simple Allometry** | 16.2 |
| **FCIM** | 14.0 |
| **Simple allometry with Fup correction** | 19.2 |
| **Overall Predicted Value** | **16.5** |

*: for human body weight of 70 kg; FCIM: Fu Corrected Intercept Method. Allometric scaling factorizing the Fup difference between human and rat.

**Figure S1.** Relationship between NOSO-502 *f*AUC_0-24_/MIC and change in bacterial burden for *E. coli* ATCC 25922 and *K. pneumoniae* 700603 in 100% MHB and 50% MHB.

**Figure S2.** Preclinical clearance versus body weight.


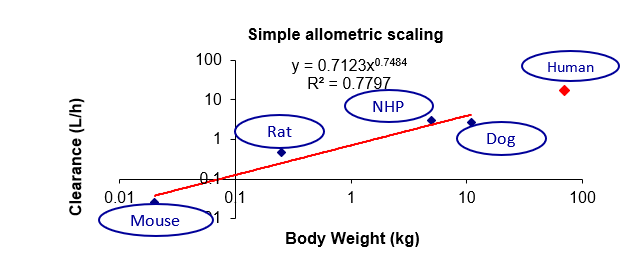


**Figure S3.** Observed versus predicted individual NOSO-502 concentrations.


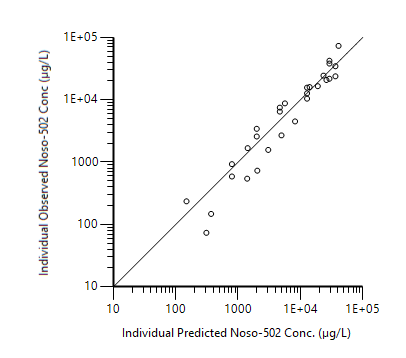

Supplement: dkag106_Supplementary_Data [file dkag106_supplementary_data.docx]
